# Understanding the Mechanisms Underlying Host Restriction of Insect-Specific Viruses

**DOI:** 10.3390/v12090964

**Published:** 2020-08-31

**Authors:** Ahmed ME Elrefaey, Rana Abdelnabi, Ana Lucia Rosales Rosas, Lanjiao Wang, Sanjay Basu, Leen Delang

**Affiliations:** 1 The Pirbright Institute, Pirbright, Woking GU24 0NF, UK; sanjay.basu@pirbright.ac.uk; 2 KU Leuven, Department of Microbiology, Immunology and Transplantation, Rega Institute for Medical Research, 3000 Leuven, Belgium; rana.abdelnabi@kuleuven.be (R.A.); analucia.rosalesrosas@student.kuleuven.be (A.L.R.R.); wang.lanjiao@kuleuven.be (L.W.)

**Keywords:** insect-specific virus, host restriction, arbovirus, mosquito-specific virus, mosquito, flavivirus

## Abstract

Arthropod-borne viruses contribute significantly to global mortality and morbidity in humans and animals. These viruses are mainly transmitted between susceptible vertebrate hosts by hematophagous arthropod vectors, especially mosquitoes. Recently, there has been substantial attention for a novel group of viruses, referred to as insect-specific viruses (ISVs) which are exclusively maintained in mosquito populations. Recent discoveries of novel insect-specific viruses over the past years generated a great interest not only in their potential use as vaccine and diagnostic platforms but also as novel biological control agents due to their ability to modulate arbovirus transmission. While arboviruses infect both vertebrate and invertebrate hosts, the replication of insect-specific viruses is restricted in vertebrates at multiple stages of virus replication. The vertebrate restriction factors include the genetic elements of ISVs (structural and non-structural genes and the untranslated terminal regions), vertebrate host factors (agonists and antagonists), and the temperature-dependent microenvironment. A better understanding of these bottlenecks is thus warranted. In this review, we explore these factors and the complex interplay between ISVs and their hosts contributing to this host restriction phenomenon.

## 1. Introduction

Arthropod-borne viruses (arboviruses) have become a serious and significant threat to human and animal health due to urban crowding, international mobility, and lack of efficient vector control programs. Generally, arboviruses are maintained by cross-species transmission between vertebrate hosts and arthropod vectors such as mosquitoes, ticks, and biting midges. Arboviruses, especially those transmitted by mosquitoes, such as dengue virus (DENV), Zika virus (ZIKV), yellow fever virus (YFV), chikungunya virus (CHIKV), and Rift Valley fever virus (RVFV) contribute significantly to disease outbreaks and epidemics in human and animal populations [1]. Over the past decade, arbovirus research was mainly focused on medically important viruses because of their pathogenicity to susceptible hosts. Vector surveillance programs have primarily been oriented to monitor circulating arboviruses of clinical and veterinary importance. Therefore, these programs surpassed a novel group of viruses referred to as insect-specific viruses (ISVs) that have been identified as a component of the insect microbiome [2]. Multiple phylogenetic studies have revealed that most ISVs are closely related to medically important arboviruses in terms of genome structure, gene order, and nucleotide sequence [3,4,5,6,7].

Unlike arboviruses, which have a dual-host tropism as they cycle between vertebrate hosts and arthropod vectors, ISVs exclusively replicate in arthropod populations, causing a persistent viral infection in the arthropod, and are vertically maintained in nature. The elucidation of the viral and host factors responsible for the limited host range of ISVs could be utilized to fully comprehend arbovirus evolution as well as to aid in the development of novel antiviral and antivectorial strategies in the fight against arboviral diseases. In this review, we will focus on ISVs identified in mosquito populations. We will also discuss, in detail, the viral and host factors contributing to ISV vertebrate restriction at different stages of the virus replication cycle.

## 2. Overview of Insect-Specific Viruses

The first ISV identified was cell-fusing agent virus (CFAV), which was isolated from an *Aedes aegypti* (*Ae. aegypti*) cell culture [8,9]. Since the discovery of CFAV, wild mosquito populations have been shown to act as a reservoir for a wide variety of ISVs, which suggests a significant heterogeneity among these viruses [10,11,12,13,14]. Thanks to the advent of next-generation sequencing applications and advanced bioinformatics tools, metagenomic studies in this scope have identified a large number of ISVs harboring wild-caught mosquitoes over wide geographical areas (reviewed in [15,16]). For instance, an RNA metaviromic study of wild-caught *Aedes aegypti* populations from Thailand and Australia suggested up to 27 ISVs may infect *Ae. aegypti* [17]. Phylogenetic analyses based on sequence identities between ISVs and other mosquito-borne viruses suggest strong evidence that ISVs may be ancestral to arboviruses [12,18]. In fact, research on ancestral trait reconstruction on ISVs of the order *Bunyavirales* has been linked to the origin of dual-host bunyaviruses in arthropod-specific progenitors [5]. Thereby, ISVs could serve as a model to study arbovirus evolution and their transition from single- to dual-host identity. ISVs have been classified within multiple different taxa, mostly in the family *Flaviviridae* and the order *Bunyavirales* (Table 1). The *Togaviridae*, *Rhabdoviridae,* and *Mesoniviridae* contain a smaller number of ISVs, as well as other taxa.

## 3. Maintenance and Replication of ISVs in Their Competent Vectors

For arboviruses, the successful establishment of a host infection is a multi-step process that demands efficient and sustained viral replication in susceptible hosts. They must overcome several bottlenecks to achieve successful infection in the arthropod vector [42]. Once an infectious blood meal is ingested by mosquitoes, the virus particles must initiate infection to midgut epithelium, disseminate into the hemocoel, spread to other organs, and reach the salivary glands. Sufficiently high viral titers should be achieved in mosquito saliva, as well as in the blood of the infected vertebrate host (if the vertebrate is not a dead-end host). On the other hand, no vertebrate hosts have been identified to support a successful replication cycle for ISVs, which raises more curiosity surrounding their maintenance in nature. Our knowledge on the maintenance of ISVs in their competent vectors is lacking. Nevertheless, some experimental studies were performed to investigate the possible transmission routes by which ISVs could be naturally maintained. The vertical transmission routes (transovarial or transovum transmission) are considered the primary means by which ISVs are maintained and propagated in their vector populations [43,44,45,46]. They have also been shown to be venereally transmitted, yet to a lesser extent [47]. In addition, ISVs such as Eilat virus (EILV) and Negev virus could be experimentally transmitted to adult mosquitoes via a high-titer of an infectious blood meal [48,49].

In recent years, there has been an increasing interest in the antiviral immune response of mosquitoes against dual-host arboviruses [50,51,52,53]. Different experimental studies were performed to investigate the delicate balance between mosquito immune response and the persistent viral infection mediated by arboviruses. Interestingly, various dual-host arboviruses have been shown to produce viral DNA forms (episomal or endogenous viral elements (EVEs)) upon mosquito infection via the reverse transcriptase activity of endogenous long terminal repeat retrotransposons [54,55,56]. These viral DNA forms are essential for mosquito survival, viral tolerance, and arbovirus dissemination and transmission in mosquitoes [57]. In contrast to arboviruses, little is known about the interaction of ISVs within their vector in terms of replication, pathogenesis, or antiviral response. The vertical transmission route of ISVs in the vector populations is able to maintain a persistent and long-lasting interaction between ISVs and their competent vectors, which, in turn, allows the integration of whole or partial sequences of ISVs in the mosquito genome [58]. Thus, these EVEs could be inherited and retained in the vector populations. Many EVEs were found closely related to the genomes of ISVs compared to these of dual-host arboviruses [59]. In general, EVEs have been demonstrated to elicit antiviral immune response by producing Piwi-interacting RNAs (piRNA), small non-coding RNAs responsible for genome integrity [60]. For example, an EVE derived from CFAV was shown to activate the piRNA pathway in persistently infected mosquitoes [61]. In addition, the small interfering (siRNA) pathway is considered the main antiviral response in mosquitoes [62]. The production of ISV-derived siRNAs has also been reported in ISV-infected mosquitoes [63,64,65,66]. In this context, both the infected cells and the virus reach a metastable equilibrium and tolerant state (persistent infection) [67]. This state, in turn, has a great impact on mosquito vector competence. Therefore, elucidating the mechanisms underlying this process might provide new antivectorial strategies to block arbovirus transmission.

Evolutionary analyses have shown that ISVs evolved and diversified within their vectors, as demonstrated by the integration of ISV segments into the mosquito genome [68,69]. Some ISVs may have evolved from single- to dual-host identity, becoming capable of infecting vertebrates on which their vector fed [6]. However, dual-host affiliated insect-specific flaviviruses (ISFs), referred to as lineage II ISFs, such as Nhumirim, Nounane, and Cháoyáng viruses have been phylogenetically grouped and suggested to have evolved from dual-host flaviviruses but lost their ability to infect vertebrate cells [70]. To understand and predict the potential of ISVs to evolve in as dual-host viruses, it is crucial to dissect the genetic factors and molecular mechanisms contributing to their replication restriction in the vertebrate host.

## 4. Host Restriction of ISVs

**Figure 1:**
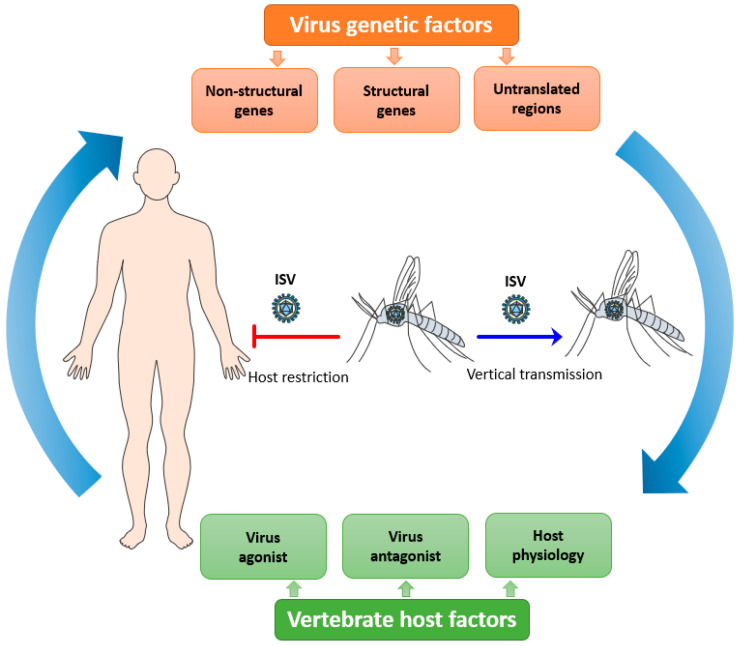
Putative overview of the host tropism of insect-specific viruses (ISVs). ISVs are maintained in mosquito populations by a vertical route of transmission. The infection and replication of ISVs are restricted in vertebrate hosts due to the complex interplay between multiple viral, host, and microenvironment factors.

Viral tropism is fundamental to consider with regards to host restriction of ISVs. It is defined as the ability of a particular virus to productively infect and replicate in a specific cell type, tissue, or species [71]. It is also an important factor that affects viral pathogenesis and disease progression in any host [71]. Viral tropism is determined by host cell susceptibility, i.e., the presence of appropriate cell receptors and/or co-receptors needed for viral attachment and entry; and host cell permissiveness, i.e., the ability of virus particles to replicate in the host intracellular environment generating new virions. For an efficient replication in the host cell, the virus should be able to interact with multiple host factors at each step of its replicative cycle and to antagonize the host immune response that hinders its replication. ISVs must overcome several integrated bottlenecks present at different levels to potentially emerge as a new dual-host virus: its genetic determinants, the vertebrate host factors, and the host microenvironment which it needs to be able to replicate (Figure 1).

### 4.1. Virus Genetic Factors

The genetic makeup of a virus is an important factor determining host susceptibility and permissiveness. For the well-studied arboviruses, limited genetic variation and quasispecies variants are observed in their virus populations when compared to other, non-arthropod-borne RNA viruses [72]. This results from the discordant demands required for efficient infection and replication in different vertebrate hosts and arthropod vectors, as the acquired mutations might increase fitness in the susceptible host but decrease it in the competent vector, or vice versa [73,74]. Adaptive mutations in these individual determinants of the arboviral genome can modulate the host and/or vector specificity [75,76,77,78]. In contrast, studies concerning ISV genetics are less abundant, although these genetic elements may play a vital role not only in host restriction but also in vector specificity.

For several ISVs, the host restriction phenomenon towards arthropods has been demonstrated at the viral genetic level [12,79,80]. A better understanding of the molecular mechanisms underlying the host restriction for several ISVs has been achieved upon the development of reverse genetics systems [24,81,82,83,84]. The generation of chimeric viruses using ISVs and arboviruses of the same taxa has been employed to document the genetic factors that may allow ISVs to cross the host barrier from mosquitoes to humans.

#### 4.1.1. Viral Structural Genes

Virus entry into the target cells is facilitated by the interaction of cellular receptor molecules and/or co-receptors with virus structural proteins. The susceptibility of vertebrate cells to arboviral infection may be primarily attributed to the conformational structure of virus particles and the presence of complement host cellular receptors. Knowledge within this area has been determined mainly for dual-host flaviviruses, whose envelope protein consists of 3 domains: I, II, and III. The domain I has a stabilizing activity for the general architecture of the envelope protein by adjoining the other two domains [85]. It has a conserved cystine residue at the N-terminus, which ISFs lack [86]. The fusion peptide, found within the extremity of domain II, has been shown to trigger a direct interaction between viral proteins and host cell membranes when the viral fusogenic conformation is encountered [87]. This peptide is made up of 14 conserved amino acids in dual-host flaviviruses, but only 8 residues are homologous in CFAV and Culex flavivirus (CXFV) [9,86]. Domain III determines virus particle internalization via a receptor-mediated endocytosis process. Multiple sequence alignment of the envelope protein of ISFs and dual-host flaviviruses identified an 8-residue sequence, which is absent in domain III of ISFs, but highly conserved in the dual-host flaviviruses [27]. ISFs also harbor four additional conserved cysteine residues in this domain. Presumably, they constitute two additional disulfide bridges that lead to a significant change in the tertiary structure of their envelope protein compared to dual-host flaviviruses [27].

The capsid proteins may play a vital role in determining the viral host range and tissue tropism [88,89]. For example, Fako virus (FAKV) is an insect-specific reovirus that was isolated from mosquitoes in Cameroon [36]. It has a simple virion architecture and minimal genetic composition, suggesting evolutionary events from more complex ancestors. It lacks one genomic segment from the 10-segment ancestor, and its core contains only one clamp protein per icosahedral asymmetric unit [36]. Moreover, the second shell from the outer shell protein is absent in FAKV’s virion. It is noteworthy that reoviruses lacking the second shell (single-shelled) are restricted to insect vectors [36]. Similarly, structural variations of the capsid proteins among members of the *Birnaviridae* family have been believed to shape their host range [90]. These notable differences are seen at the spike−spike contacts between adjacent trimers in the T = 13 lattice [90]. For instance, substantial structural differences at this interface have been reported between Espirito Santo virus (ESV), an insect-specific virus of the *Birnaviridae* family, and the relevant vertebrate-infecting viruses of the same family [31]. Of note, ESV belongs to the *Entomobirnavirus* genus, whose members replicate exclusively in insects [91]. Therefore, additional studies may be warranted to unravel the impact of structural disparities of the capsid proteins on viral architecture which, in turn, fabricate the virus−host range.

Reverse genetics is a relevant approach to study the individual role and function of ISV genes. For example, a chimeric YFV containing the envelope proteins of Niénokoué virus (NIEV), an ISF, (NIEV/YFV) was not able to infect vertebrate cells; albeit, it contained the dual-host replicative proteins [24]. Binjari virus (BinJV) is another example of an ISF with restricted replication in vertebrate cells. A West Nile virus (WNV) chimera carrying BinJV prME proteins (WNV/BinJV_prME_) showed limited viral infection in vertebrate cells, showing that BinJV structural proteins could facilitate vertebrate infection, albeit inefficient [20]. Another example is EILV, an insect-specific alphavirus related to the Western equine encephalitis virus serocomplex in the *Alphavirus* genus. While EILV is entry deficient in vertebrate cells, the chimeric (EILV/CHIKV), composed of EILV encoding structural proteins of CHIKV, a dual-host alphavirus, was able to bind and enter these cells [82]. Moreover, the chimera of SINV encoding the structural proteins of EILV (SINV/EILV) displayed a low infection efficiency in vertebrate cells that increased upon RNA transfection, which supports that ISV host restriction is present at the entry step [92].

These examples indicate that some ISVs are not capable of infecting vertebrate cells due to the lack of the appropriate envelope protein structure needed for virion attachment and the absence of the orchestrated conformational change required for membrane fusion. However, there are also examples suggesting that the structural genes are not sufficient to explain the host restriction of intermediate-host viruses. For instance, Bamaga virus (BgV) is an intermediate flavivirus between ISFs and dual-host flaviviruses of the YFV group [80]. BgV was shown to be restricted in vivo and in multiple vertebrate cells except for opossum cells, a marsupial derived cell line [80]. Interestingly, BgV was not able to replicate in multiple vertebrate cells even though its prME proteins were replaced with these of WNV, while the reciprocal chimera (WNV/BgV_prME_) showed viral replication in all vertebrate cells [93]. This suggests that the host restriction of BgV may be attributed to other genetic elements rather than the structural proteins.

#### 4.1.2. Viral Non-Structural Genes

The viral non-structural proteins include different enzymes and co-factors needed for a complete replication cycle in the infected host, and they are also suggested to contribute to host restriction of ISVs. For example, chimeric variants of the Palm Creek virus (PCV), an ISF, carrying the prME proteins of dual-host flaviviruses (PCV/WNV_prME_, PCV/DENV_prME_, PCV/ZIKV_prME_), failed to initiate a reproductive replication cycle in vertebrate cells, although the chimeric viruses were likely able to enter the cells [83]. Moreover, limited to no viral replication has been reported when multiple vertebrate cell lines were infected with the chimeric BinJV carrying prME proteins of WNV (BinJV/WNV_prME_) [20]. Furthermore, the chimeric EILV carrying the structural proteins of SINV (EILV/SINV) was completely unable to replicate into vertebrate cells [92]. This finding was further supported by in vivo infection studies performed in newborn mice [92]. The above observations suggest that the structural proteins of dual-host viruses are not sufficient to rescue the replication of ISVs in vertebrate cells, and the non-structural proteins are also involved in host restriction. The adaptation of the intermediate-host virus, BgV, to vertebrate cells at 37 °C demonstrated the involvement of the non-structural proteins NS4A-B and NS5 in the host restriction of BgV [93]. The identified mutations, occurring close to viral protease cleavage sites (NS4A/2K and NS4B/NS5) of BgV, played a critical role in viral infectivity and pathogenesis in vivo [93].

In addition, multiple ISFs have shown conserved deletions in the NS5 methyltransferase (MTase) domain [27], located at the N-terminus of the non-structural NS5 protein. In contrast, the (residues 39–52) deletion was not present in dual-host flaviviruses. The NS5 MTase enzyme is responsible for guanine N-7 and ribose 2′-OH methylation during flavivirus 5′cap formation [94]. The MTase domain harbors the αA3-motif (residues 36–51) [95]. When mutations were inserted in this motif, most mutations did not alter WNV replication; however, a single mutation (E46L) significantly inhibited virus replication in vertebrate cells [95]. Thus, it would be compelling to investigate the impact of this MTase motif on virus replication in both mosquito and mammalian cells.

Nidoviral uridylate-specific endoribonuclease (NendoU/nsp15) was formerly considered one of the highly conserved domains and genetic markers in nidoviruses [96]. This domain is responsible for cleaving both the single- and double-stranded RNAs at the 3′ uridylate site [97]. Despite the vital role of NendoU in viral RNA synthesis, it has been reported that the presence of this domain is restricted to vertebrate-infecting nidoviruses (*Coronaviridae*, *Arteviridae*, and *Roniviridae*) [98,99]. For instance, members of the invertebrate-infecting nidoviruses (*Mesoniviridae*) such as Cavally virus, Nam Dinh virus, Hana virus, Méno virus, and Nsé virus, have been shown to lack NendoU domain [33,100,101]. Identification and characterization of these insect-specific mesoniviruses also presented an appropriate model to fill the missing evolutionary gap in our endeavors to understand the emergence of nidoviruses [33]. Recently, the NendoU domain has been demonstrated to mediate an immunomodulatory function by suppressing the innate immune system and evasion of the host dsRNA sensors in vertebrate cells [102,103]. Thus, the role and impact of the NendoU domain in restricting the host range of ISVs should be further investigated.

An interesting model virus is Rabensburg virus (RABV), a mosquito-specific flavivirus related to WNV [104]. RABV is unable to infect several mammalian cells [105], but was recently shown to infect avian cells reaching a high titer, despite the fact that RABV has not been isolated from birds or other vertebrate hosts in nature [106]. As a result of these unique characteristics, RABV is considered an intermediate between ISFs and the dual-host flaviviruses of the Japanese encephalitis virus complex [105]. Infectious virus particles were released when HEK293 cells were transfected with RABV RNA, demonstrating that the RABV non-structural proteins are replication competent and a possible bottleneck at the entry level [105,106]. Of note, RABV has been shown to replicate in vertebrate cells at temperatures below 35 °C, albeit inefficiently [18]. Adaptation of RABV for replication in these cells at 35 °C resulted in multiple non-synonymous mutations mainly identified in the non-structural proteins [106]. Especially the conserved mutation in the helicase domain of non-structural protein 3 may be one of the driving forces for a successful switch of RABV to a vertebrate host [106]. Thus, RABV may provide an adequate model to elucidate the genetic changes underlying arbovirus replication in vertebrate cells, which promotes host switching.

It would also be of great interest to investigate the host restriction features for viruses that lay at the boundary between insect-specific and dual-host identities. For instance, Caainguá virus, a novel alphavirus closely related to the equine encephalitis complexes, could be considered as an ISV based on its vertebrate restriction characteristic; however, its replication in human mononuclear cells negates this speculation [107]. Therefore, understanding these mechanisms of host switching will not only explain arbovirus evolution but also the evolutionary arms race undertaken by viruses and their vertebrate host.

#### 4.1.3. Viral Untranslated Regions (UTRs)

The 5′ and 3′ UTRs flanking the coding region of the alphavirus and flavivirus genomes are vital elements for optimal virus replication and immune modulation. The highly structured 3′UTR is characterized by short sequence repetitions named dumbbell (DB) and stem-loop (SL) structures and is the source of a subgenomic flavivirus RNA with antagonizing type I interferon activity [108]. Within the flaviviruses, conserved DB and SL structural blocks are present in dual-host flaviviruses, whereas they are absent in the ISFs CFAV and CxFV [109]. It has been hypothesized that duplicated DB and SL structures have a high potential for high-order interactions and are required for dual-host flaviviruses to maintain high virus fitness during host−vector switching [110,111]. Besides, the mutations in DENV SL acquired during replication in mosquitoes have a detrimental impact on viral replication in mammalian cells [111]. The 3′UTR of certain ISFs, such as CFAV and Aedes flavivirus, conserve multiple direct sequence repeats, but they lack the potential for high-order interactions. In contrast, ISFs such as Cháoyáng virus and Nhumirim virus have a single copy of DB structure in their 3′UTR [110,111]. Thus, these conserved repeated elements are potentially a common feature among mosquito-borne flaviviruses. Junglen, et al. generated a chimeric NIEV carrying the YFV 3′UTR (NIEV/YFV_3′UTR_) to test this hypothesis [24]. Although (NIEV/YFV_3′UTR_) was unable to infect vertebrate cells, its translation efficiency was increased after RNA electroporation [24]. This demonstrates that the 3′UTR is not a major determinant for the host restriction of ISVs in vertebrate cells.

In the case of alphaviruses, newly emerged CHIKV variants enriched with direct repeats at its 3′UTR exhibited a high replication rate and fitness in mosquito cells, whereas direct repeat-deletion mutants were positively selected in mammalian cells [112,113]. These findings were evaluated on vertebrate cells infected with the chimeric EILV carrying SINV 3′UTR, yet no viral infection was observed [92]. This confirmed that dual-host alphavirus 3′UTR elements were not able to rescue EILV replication in mammalian cells, suggesting that the 3′UTR is not a central determinant for the restriction of insect-specific alphavirus in vertebrates.

### 4.2. Vertebrate Host Factors

Several host cellular receptors involved in arbovirus attachment and entry have been identified in mammalian cells [114,115], and concurrently their orthologs in mosquito cells [116,117]. Following virus internalization by the host cell, the virus hijacks the host cellular machinery to facilitate its intracellular trafficking and genome translation and replication. Host factors encountered by viruses could be divided into two main categories: virus-agonist factors, needed for the virus to complete its replicative cycle in the infected cells; and virus-antagonist factors, which interfere with the virus replicative cycle, suppressing and/or limiting viral infection and propagation.

#### 4.2.1. Virus-Agonist Factors

Genome-wide approaches involving transcriptomic and proteomic analyses have demonstrated that arboviruses rely on several host proteins to complete their replication cycle in their vectors and susceptible hosts [118,119,120,121,122,123,124,125]. Likewise, ISVs use proteins from arthropod cells to replicate. In vertebrate cells, ISV replication might be confronted with the absence of required cellular factors. For instance, divergent and specific host factor dependencies have been demonstrated in viruses even though they are stemming from the same family [123]. RNA of the chimeric (NIEV_prME_/YFV) was not able to replicate in vertebrate cells, and no viral progeny was assembled or released, although the chimeric RNA was translated after transfection [24]. Thus, it might be plausible that the lack of certain host factors in vertebrates or the structural divergence from their mosquito orthologs contributes to the vertebrate restriction phenomenon of ISVs. Of note, the inability of Modoc virus, a member of the no-known vector group of the *Flavivirus* genus [126], to replicate in mosquito cells has been attributed to the downstream post-entry stage, where virus-agonist cellular factors play an important role [127,128,129]. All chimeric viruses used to study ISV host restriction have been constructed thus far between ISVs and dual-host viruses. The generation of chimeras between ISVs and no-known vector members may shed some light on the vector range of emerging flaviviruses and their phylogenetic relatedness. Therefore, ISVs may represent the missing evolutionary link between dual-host and vertebrate-only viruses.

#### 4.2.2. Virus-Antagonist Factors

Contrarily to the virus-agonist factors, virus-antagonist factors inhibit ISV infection and replication in vertebrates. These factors are mainly related to the innate immune response, which is the first line of defense against viral infection [130,131]. In general, pattern-recognition receptors (PRRs), in both vertebrates and invertebrates, serve as sentinels for viral infection as they are engaged in detecting viral DNA, RNA, and dsRNA-replicative forms. These elements are referred to as pathogen-associated molecular patterns, which are produced in virus-infected cells [132]. While the conserved insect innate immune response to viral infection includes RNA interference, Toll, immune deficiency factor, and Janus kinase-signal transduction and activators of transcriptions (JAK/STAT) pathways [52], the innate immune response is less conserved between insects and vertebrates. For instance, interferons (IFNs) are key contributors to the mammalian JAK-STAT pathway to provide an effective antiviral response, but their orthologs have not been identified in insects [133].

Multiple ISVs have shown to lack the ability to infect the vertebrate Vero cell line [13,35,134,135,136,137,138,139], although Vero cells are deficient in interferon production [140]. PCV, PCV carrying WNV prME (PCV/WNV_prME_), or the reciprocal chimera (WNV/PCV_prME_) were unable to replicate in IFN-α/β receptor knockout (IFNAR^−/−^) mouse embryonic fibroblasts (MEFs) [83]. Similar results were obtained with the chimeric PCV carrying prME of ZIKV or DENV [141]. Moreover, Parramatta River virus, an ISF, failed to replicate in IFNAR^−/−^ MEFs even after bypassing the viral entry step [83]. Furthermore, Gouléako goukovirus and Herbert herbevirus, ISVs of the *Bunyavirales* order, displayed no viral growth in MEFs lacking MDA5 or RIG-I, intracellular PRRs that induce IFN-α/β production upon dsRNA sensing [30,131,134]. Recently, limited to no viral replication was observed in MEF cells lacking IFNAR or RNase L, when they were infected with BinJV, excluding the involvement of these pathways as a major bottleneck in host restriction [20,93]. In addition, the BinJV chimera carrying prME proteins of ZIKV (BinJV/ZIKV_prmE_) or the chimeric EILV containing the structural proteins of CHIKV (EILV/CHIKV) failed to replicate in immunocompromised mice, demonstrating their incompetence to adapt a vertebrate replicative phenotype [82,84]. These results indicate that the IFN-mediated response is not the primary barrier to ISV replication in vertebrate cells. Possibly, they encounter an IFN-independent antiviral response in vertebrates.

In contrast to the aforementioned ISVs of the *Bunyavirales* order, knocking down RIG-I and MDA5 increased the replication of the ISF Kamiti river virus (KRV) in vertebrate cells [79]. Of note, interferon regulatory factors (IRF) play an essential role in IFN-α/β induction once RIG-I and MDA5 recognize viral products [131]. Knocking out IRFs (3, 5, 7) in MEFs allowed KRV to complete its replication cycle, shedding trace amounts of infectious virions for only 3 days post-infection [79]. On the one hand, it could be that the antiviral immune response encountered by KRV is largely mediated by IFN-independent signaling, which depends on IRF3 expression [142,143]. On the other hand, perhaps KRV can partially evade the innate immune system, similar to pathogenic flaviviruses, based on its non-structural proteins or 3′UTR. It has been described that KRV has a 3′UTR (1208 nt) twice as long as that of CFAV (556 nt) and other flaviviruses (400–700 nt) [10], which arose from a duplication of a primordial KRV 3′UTR [144]. Unlike other ISFs, the 3′UTR of KRV consists of two copies of SL structure [145]. It has been reported that the deletion of this SL structure in DENV (ΔSL-DENV) resulted in decreased fitness in vertebrate cells [109]. Flavivirus 3′UTR duplication has been suggested as an evolutionary trait to maintain high viral fitness in vertebrate cells, despite the mosquito-associated mutations in this region generated during viral replication [146]. This trait might explain how the multigenic determinants of KRV, in contrast to those of other ISVs, supported its replication in immunocompromised vertebrate cells.

#### 4.2.3. Microenvironment Affecting Host Physiology

Besides arthropod genotype and virus strain, the (host body) temperature is a crucial parameter for the transmission of arthropod-borne pathogens because it affects the extrinsic incubation period [147,148,149]. Mosquitoes such as *Ae. aegypti* can complete their life cycle at a temperature range from 15 to 37 °C [150]. Arboviruses can replicate in vertebrate hosts at high temperatures (36.5–42 °C), whereas both ISVs and arboviruses replicate at ambient temperatures, around 28 °C, in their associated vector [5]. For instance, the dual-host ZIKV could be transmitted by *Ae. aegypti* across the whole temperature range of 22.7–34.7 °C [151]. Similarly, Rift Valley fever virus and La Crosse virus, pathogenic viruses of the order *Bunyavirales*, were replication competent in mosquito cells at temperatures ranging from 29–34 °C. However, insect-specific viruses of the same order, such as Jonchet orthojonvirus, Ferak orthoferavirus, Gouléako goukovirus, and Herbert herbivirus, exhibited impaired replication at 32 °C compared with that at 28 °C and a complete inhibition above 33 °C [5]. Dianke virus and Agua salud alphavirus, which belong to the *Alphamesonivirus 1* and *Alphavirus* genera, respectively, showed reduced viral replication in mosquito cells at elevated temperatures [35].

BinJV failed to replicate in vertebrate cells at 37 °C, but low levels of BinJV replication were detected upon infection under high MOI at 34 °C [20]. These results add temperature dependence together with the previously mentioned BinJV genetic determinants as the main bottlenecks involved in its host restriction. The intermediate host virus BgV has been demonstrated to replicate in a subset of vertebrate cells at 28 °C or 37 °C. However, its replication was host restricted in other vertebrate cells at both of these temperatures [93]. Interestingly, BgV host restriction was shown to be temperature-dependent as it replicated in multiple vertebrate cells at 34 °C, including those that did not support its replication at 37 °C [80,93]. In this light, it is noteworthy to mention that enhanced CHIKV replication in vertebrate cells at a lower temperature (22 °C) is likely attributed to a reduced type I interferon response [152]. Together, these examples highlight that the replication of ISVs and intermediate-host viruses is temperature-dependent, and tolerating these elevated temperatures might co-facilitate their transition from single- to dual-host identity.

## 5. Conclusions

The discovery of a wide variety of ISVs within the mosquito microbiome led to the suggestion that some insect-specific viruses over time gained the ability to expand their tropism from a single host to dual hosts [6]. To this end, ISVs must overcome different bottlenecks encountered in the ecologically complex triad of the vector, vertebrate host, and the temperature-dependent microenvironment [18]. Dissecting these factors will further enlarge our understanding and preparedness for the emergence of new arboviruses. The mechanisms by which viruses can expand their host range are still largely unknown, and further studies are required to clarify to what extent the genetic properties of ISVs are contributing to vertebrate restriction. Thus, elucidating these determinants will not only advance our knowledge on ISV vertebrate restriction but also provide insight into the adaptive and virulence factors utilized by arboviruses in comparison to ISVs, which might be helpful to develop novel strategies and interventions against arboviral diseases.

Several reports postulated the beneficial deployment of ISVs as a novel biocontrol weapon by suppressing or limiting the transmission of medically important arboviruses [15,153,154,155]. They are also utilized as platforms for diagnostics and vaccine development based on their vertebrate restriction feature [84,156]. Thus, ISVs are considered a valuable tool that could be fabricated to serve our endeavors in the fight against arboviruses.

## Figures and Tables

**Table 1:** Classification of the most relevant insect-specific viruses with confirmed mosquito vector species.

Taxa	Genus	ISV	Host ^1^	Reference
*Flaviviridae*	*Flavivirus*	Aedes flavivirus	*Aedes* spp.	[19]
		Binjari virus	*Aedes normanensis*	[20]
		Cell fusing agent virus	*Aedes* spp.	[8]
		Cháoyáng virus	*Aedes* spp. *Culex pipiens* *Armigeres subalbatus*	[21,22]
		Culex flavivirus	*Culex* spp.	[23]
		Kamiti River virus	*Aedes macintoshi*	[10]
		Niénokoué virus	*Culex* spp.	[24]
		Nhumirim virus	*Culex chidesteri*	[25]
		Nounané virus	*Uranotaenia mashonaensis*	[26]
		Palm Creek virus	*Coquillettidia xanthogaster*	[11]
		Parramatta River virus	*Aedes vigilax*	[27]
*Bunyavirales*	*Phasivirus*	Badu phasivirus	*Culex* spp.	[28]
		Phasi Charoen-like phasivirus	*Aedes aegypti*	[29]
	*Orthoferavirus*	Ferak orthoferavirus	*Culex decens*	[5]
	*Goukovirus*	Gouléako goukovirus	*Anopheles* spp. *Culex* spp. *Uranotaenia* spp.	[13]
	*Herbevirus*	Herbert herbevirus	*Culex nebulosus*	[30]
	*Orthojonvirus*	Jonchet orthojonvirus	*Culex* spp.	[5]
*Birnaviridae*	*Entomobirnavirus*	Espirito Santo virus	*N/A*	[31]
*Mesoniviridae*	*Alphamesonivirus 1*	Cavally virus	*Aedes* spp. *Anopheles* spp. *Culex* spp. *Uranotaenia* spp.	[32]
		Nam Dinh virus	*Culex* spp. *Aedes albopictus*	[33,34]
		Dianke virus	*Aedes* spp. *Anopheles* spp. *Culex* spp. *Mansonia* spp. *Uranotaenia* spp. ceratopogonids	[35]
*Reoviridae*	*Dinovernavirus*	Fako virus	*Aedes* spp. *Eretmapodites* spp.	[36]
*Rhabdoviridae*	*Almendravirus*	Arboretum almendavirus	*Ochlerotatus* *fulvus*	[37]
		Puerto Almendras almendavirus	*Psorophora albigenu*	[37]
	*Mousrhavirus*	Moussa Mousrhavirus	*Culex decens*	[38]
*Togaviridae*	*Alphavirus*	Agua Salud alphavirus	*Culex declarator*	[39]
		Eilat virus	*Anopheles coustani*	[12]
		Tai Forest alphavirus	*Culex decens*	[40]
		Yada yada virus	*N/A*	[41]

^1^ The host range of insect-specific viruses identified up to date. *N/A* the vector species of ISV are not determined yet.
